# Optoelectronic Properties of a Cylindrical Core/Shell Nanowire: Effect of Quantum Confinement and Magnetic Field

**DOI:** 10.3390/nano13081334

**Published:** 2023-04-11

**Authors:** Mohamed El-Yadri, Jawad El Hamdaoui, Noreddine Aghoutane, Laura M. Pérez, Sotirios Baskoutas, David Laroze, Pablo Díaz, El Mustapha Feddi

**Affiliations:** 1 Group of Optoelectronic of Semiconductors and Nanomaterials, ENSAM, Mohammed V University in Rabat, Rabat 10100, Morocco; 2 Instituto de Alta Investigación, CEDENNA, Universidad de Tarapacá, Casilla 7D, Arica 1000000, Chile; 3 Departamento de Física, FACI, Universidad de Tarapacá, Casilla 7D, Arica 1000000, Chile; 4 Department of Materials Science, University of Patras, GR-26504 Patras, Greece; 5 Departamento de Ciencias Físicas, Universidad de La Frontera, Casilla 54-D, Temuco 4780000, Chile; 6 Institute of Applied Physics, Mohammed VI Polytechnic University, Lot 660, Hay Moulay Rachid Ben Guerir, Ben Guerir 43150, Morocco

**Keywords:** core/shell, nanowire, magnetic field, donor impurity, binding energy, photoionization cross-section

## Abstract

This study investigates the effect of quantum size and an external magnetic field on the optoelectronic properties of a cylindrical Al${}_{x}$Ga${}_{1-x}$As/GaAs-based core/shell nanowire. We used the one-band effective mass model to describe the Hamiltonian of an interacting electron-donor impurity system and employed two numerical methods to calculate the ground state energies: the variational and finite element methods. With the finite confinement barrier at the interface between the core and the shell, the cylindrical symmetry of the system revealed proper transcendental equations, leading to the concept of the threshold core radius. Our results show that the optoelectronic properties of the structure strongly depend on core/shell sizes and the strength of the external magnetic field. We found that the maximum probability of finding the electron occurs in either the core or the shell region, depending on the value of the threshold core radius. This threshold radius separates two regions where physical behaviors undergo changes and the applied magnetic field acts as an additional confinement.

## 1. Introduction

In view of the development of new tools for elaboration, observation and analysis of new architectural nanostructures, nanosciences and nanotechnologies have experienced dazzling advances so far. Among all types of nanomaterials, one-dimensional (1D) nanostructures display novel sensing and light harvesting applications [1,2,3,4,5,6,7]. The progress of energy storage and conversion devices have been enabled by surface and architectural engineering of the chemical and physical properties of 1D materials [8,9,10,11]. Semiconductor nanowires (NWs) have also found a place in biology and medicine. Indeed, nanoelectronic devices based on 1D Si nanowires that have been employed to detect biomolecules, biomarkers and viruses [12,13] exhibited remarkable performance in bioelectronic and drug delivery applications [14,15]. Nanotechnologies—formalization of the concepts and processes of nanosciences in applications—already have broad fields of application with 1D materials and a set of prospective fields will undoubtedly have major benefits and new functional properties in the years to come; by increasing the possibilities in the synthesis of these structures with distinct types of materials to yield complex compositions.

With recent advances in material manipulation at the nanoscale, the size and shape of nanostructures can be controlled to produce new architectural structures, such as the so-called core/shell structures. These structures provide alternative forms of charge carrier quantum confinement. Among the first colloidal type-II systems studied were CdTe/CdSe core-shell particles [16]. In these structures, the band offsets of the two materials cause the electron to be located in the CdSe shell, while the hole is confined to the core of the nanocrystals. However, the first successful attempts to grow Type-I core-shell systems were for CdSe/ZnS [17,18] and soon after for CdSe/CdS [19]. In this case, the band alignment leads to confinement of the charge carriers in one side only, either inside the core or the shell. Today, a variety of Type-I and Type-II core-shell structures can be synthesized from group III–V or II–VI bulk semiconductors. In particular, core-shell nanowires have been the subject of interest for many researchers from an experimental point of view. These structures can be readily combined with properly designed metallic components and serve as key building blocks for unique photonic and optoelectronic nanodevices with unprecedented optical functions [20]. Furthermore, they can be synthesized with varying core and shell diameters [21]. Recently, more studies have investigated the development of core-shell nanowires using different processes [22,23,24,25,26].

Theoretical studies of nanostructures involve the use of computational models and simulations to understand the properties and behavior of nanoscale materials. These studies are important for guiding the design and development of materials with specific properties tailored for particular applications. They can provide a deeper understanding of the nanoscale phenomena that occur in materials and help to guide the development of materials with enhanced properties and functionalities. The size and shape effects of nanostructures result in quantization of electronic structures and resultant physical properties that differ significantly from those of bulk systems. Nowadays, some studies are being conducted on core/shell NWs to reveal their unique physical properties. Using the Monte Carlo approach, Dan et al. have investigated the magnetic and thermodynamic properties of a cylindrical ferrimagnetic Ising nanowire system with core/shell structure [27]. Within the first-principles calculations, S.U. Rehman et al. have studied the optoelectronic properties of ZnS NWs and Zn/Si core/shell NWs oriented along different directions [28]. They have shown that the electrical and optical properties are strongly dependent on the orientation of the structures. J. Zheng et al. have reported a theoretical description of the electronic and optical properties of GaN/AlN core/shell NWs [29]. They have demonstrated that these properties are sensitive to the core and shell sizes. By numerical solution of the Schrödinger equation within the effective mass approximation, M. Kouhi has theoretically investigated the effect of incident light intensity, relaxation time, core radius and shell thickness on linear, nonlinear and total optical absorption coefficients and refractive index changes in GaN/AlGaN core/shell NWs [30]. In addition, doping materials is an important technique to tailor their properties for specific applications. In the case of core/shell NWs, the doping process can be carried out by introducing impurities in either the core or the shell material. For example, doping the core material can change the electrical properties of the core and thus affect the overall properties of the nanowire. Similarly, doping the shell material can modify the optical properties of the nanowire [31,32,33]. Despite the theoretical research that has been carried out so far, it remains insufficient to unveil all the fascinating properties of the core/shell NWs found by experiments.

This paper presents a theoretical description of the electronic and optical properties of cylindrical core/shell NWs. To determine the electron ground state energy inside this structure, we shall adopt the one-band effective mass model from which the coupling between conduction and valence band is disregarded. Within an appropriate trial wave function, we shall calculate the electron ground state energy under an applied external magnetic field using the variational method. We shall also analyze the interaction between the electron and a donor impurity assumed to be localized in the core region, through the calculation of the binding energy and the photoionization cross-section. The organization of this work is as follows: the generalized electron-impurity Hamiltonian and mathematical formulas to compute the electronic and optical properties are given in Section 2. Physical parameters used in calculation and results are reported in Section 3. Then, the physical properties in question are discussed in Section 4. Finally, the summary of this work is presented in Section 4.

## 2. Background Theory

**Figure 1:**
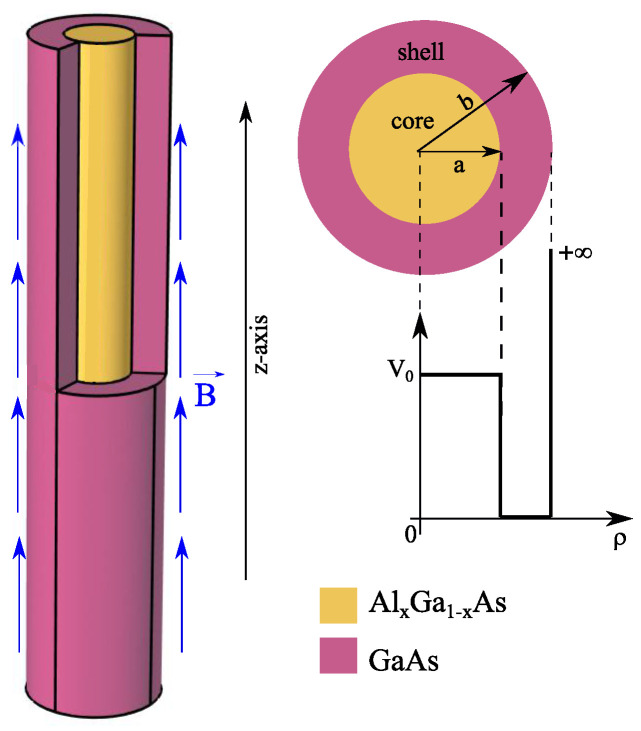
Schematic illustration of the cylindrical core/shell nanowire and the corresponding conduction band structure. The core is taken to be Al${}_{x}$Ga${}_{1-x}$As material with controllable radius *a*, surrounded by GaAs material-based shell with radius *b*. we assume that the structure is under the effect of an external magnetic field $\vec{B}$ along the *z*-axis.

The system under consideration is a core/shell nanowire with cylindrical symmetry, containing a confined electron-donor impurity system. Due to the cylindrical symmetry, the degree of freedom of the electron is reduced from 3 to 1. We assume that the core material is made of Al${}_{x}$Ga${}_{1-x}$As with a radius of *a*, covered by GaAs shell material with a radius of *b*. The core/shell structure is embedded into a matrix material with a wide band gap. As a result of the difference in band gap between the core and shell materials, we can consider the potential barrier to be finite in the core region and infinite in the exterior region. Figure 1 provides a schematic illustration of our model and the corresponding conduction band structure. Within the framework of the effective mass approximation, the Hamiltonian governing the electron-donor impurity system in the presence of an external magnetic field along the *z*-axis is given by:

(1) $$H_{eD}=\frac{1}{2m_{e}^{*}\left(i\right)}{\left(\vec{P}-e\vec{A}\right)}^{2}-\frac{e^{2}}{\varepsilon_{i}\left|{\vec{r}}_{e}-{\vec{r}}_{D}\right|}+V_{e}^{i}\left(r_{e}\right),\;\;\left(i=core,shell\right)$$

The averaged interaction of the electron with host atoms and other electrons is felt as if it has an effective mass $m_{e}^{*}$, which properly depends on each material and strongly differs from the electron’s rest mass in vacuum $m_{0}$. $\vec{P}=-i\hbar \vec{\nabla }$ and $\vec{A}$ represent the momentum operator and the vector potential, respectively. The Coulomb interaction between the electron and donor impurity is expressed as a function of the material dielectric constant $\varepsilon_{i}$ and the distance electron-impurity, which is the difference between the electron and the impurity positions ${\vec{r}}_{e}$ and ${\vec{r}}_{D}$, respectively. We assume that during the motion the electron undergoes a square confinement potential. With regard to the nanowire (2D confinement), it is evident that $V_{e}^{i}\left(r_{e}\right)$ will be strongly pronounced in the lateral direction of the cylinder (ρ). So that for a best solution of the Schrödinger equation, our approach involves modeling it as the sum of an infinite square well along the z-direction with a large size *h* (the height of the core/shell) and a radial confinement expressed as:

(2) $$V_{e}\left(\rho_{e}\right)=\left\{\begin{array}{c} V_{e}^{core}=V_{0},\;\mathrm{for}\;0<\rho_{e}<a\\ V_{e}^{shell}=0,\;\mathrm{for}\;a<\rho_{e}<b\\ \infty \;\;\mathrm{Otherwise}. \end{array}\right.$$

where $V_{0}$ is the potential barrier between the core and shell regions. For a uniform magnetic field, we can write $\vec{A}\left({\vec{r}}_{e}\right)=\frac{1}{2}\vec{B}\wedge {\vec{r}}_{e}$. Using the cylindrical coordinates system, the effective Bohr radius $a_{D}^{*}=\frac{\hbar^{2}\varepsilon_{shell}}{m_{e}^{*}e^{2}}$ as unit for length and $R_{D}^{*}=\frac{e^{2}}{2\varepsilon_{shell}a_{D}^{*}}$ as unit for energy, we can rewrite the Hamiltonian as follows:

(3) $$H_{eD}=\left\{\begin{array}{c} -\mu \nabla^{2}-\frac{\varepsilon_{shell}}{\varepsilon_{core}}\frac{2}{|{\vec{r}}_{e}-{\vec{r}}_{D}|}+\mu \gamma L_{z}+\frac{\mu }{4}\gamma^{2}\rho_{e}^{2}+V_{0},\;\mathrm{if}\;0<\rho_{e}<a\\ -\nabla^{2}-\frac{2}{|{\vec{r}}_{e}-{\vec{r}}_{D}|}+\gamma L_{z}+\frac{1}{4}\gamma^{2}\rho_{e}^{2},\;\mathrm{if}\;a<\rho_{e}<b \end{array}\right.$$

where $\mu =\frac{m_{shell}^{*}}{m_{core}^{*}}$ is the effective mass ratio, $\gamma =\frac{\hbar \omega_{c}}{R_{D}^{*}}$ is a dimensionless measure of the magnetic field where $\omega_{c}=\frac{eB}{m_{shell}^{*}}$ is the cyclotron frequency of the electron and $L_{z}$ is the z-component of the angular momentum in units of *ℏ*.

The exact solution of Schrödinger equation $H_{eD}\psi_{eD}\left({\vec{r}}_{eD}\right)=E_{eD}\left(B\right)\psi_{eD}\left({\vec{r}}_{eD}\right)$ seems impossible; therefore, the variational approach will be the approved method in this work. We assume that the wave function $\psi_{eD}\left({\vec{r}}_{eD}\right)$ can be written as the production of the three functions $f(\rho_{e})$, $h(\varphi_{e})$ and $g(z_{e})$ describing the state of the electron without impurity, with two other functions satisfying the magnetic perturbation and the Coulomb interaction with donor impurity, which are $exp(-\lambda_{1}\rho_{e}^{2})$ and $exp(-\lambda_{2}r_{eD})$, respectively. With $r_{eD}=\sqrt{{\left(\rho_{e}-\rho_{D}\right)}^{2}+{\left(z_{e}-z_{D}\right)}^{2}}$, $\lambda_{1}$ and $\lambda_{2}$ are variational parameters.

The boundary and continuity equations for the wave function and its derivative must be ensured to determine the electron energies. In addition, the wave function must be finite for $\rho_{e}$→0 to fulfill the normalization condition. After detailed calculations, the electron wave function by rotation of angle $\varphi_{e}$ around the growth axis is $h\left(\varphi_{e}\right)=e^{im\varphi_{e}}$ and along the *z*-axis $g\left(z_{e}\right)=e^{ik_{z_{e}}z_{e}}$. Note that the solution of the Schrödinger equation along the radial direction requires to consider two possible cases, $E_{e\rho }<V_{0}$ and $E_{e\rho }>V_{0}$, leading to the following ordinary and modified Bessel functions:

- For $E_{e\rho }<V_{0}$
  (4) $$f\left(\rho_{e}\right)=\left\{\begin{array}{c} A_{1}I_{m}\left(\sqrt{\frac{V_{0}-E_{e\rho }}{\mu }}\rho_{e}\right),\;\mathrm{if}\;0<\rho_{e}<a\\ B_{1}J_{m}\left(\sqrt{E_{e\rho }}\rho_{e}\right)+Y_{m}\left(\sqrt{E_{e\rho }}\rho_{e}\right),\;\mathrm{if}\;a<\rho_{e}<b. \end{array}\right.$$
- For $E_{e\rho }>V_{0}$
  (5) $$f\left(\rho_{e}\right)=\left\{\begin{array}{c} A_{2}J_{m}\left(\sqrt{\frac{E_{e\rho }-V_{0}}{\mu }}\rho_{e}\right),\;\mathrm{if}\;0<\rho_{e}<a\\ B_{2}J_{m}\left(\sqrt{E_{e\rho }}\rho_{e}\right)+Y_{m}\left(\sqrt{E_{e\rho }}\rho_{e}\right),\;\mathrm{if}\;a<\rho_{e}<b. \end{array}\right.$$

m=0,±1,… is the Bessel function order on one side and the magnetic quantum number on the other, since it comes from the term $L_{z}$ of the Hamiltonian. $I_{m}$ is the modified Bessel function of the first kind, $J_{m}$ and $Y_{m}$ are the ordinary Bessel function of the first and second kind [34]. $A_{1}$, $B_{1}$, $A_{2}$ and $B_{2}$ are constants to be determined. First, we can write the total energy of the electron without taking into account the influence of the magnetic field and the presence of the donor impurity as:

(6) $$E_{e}=E_{e\rho }=E_{n,m}$$

It is obvious that this energy depends on the proper quantum numbers *m* and *n*, which denote the Bessel function order and the $n^{th}$ solution of the transcendental derived from the boundary conditions, respectively. In particular, the ground state energy corresponds to n=1,m=0. Therefore, the energy $E_{e\rho }=E_{1,0}$ satisfies the following equation:

- For $E_{e\rho }<V_{0}$
  (7) $$A_{1}\sqrt{\mu \left(\frac{V_{0}}{E_{e\rho }}-1\right)}I_{1}\left(\sqrt{\frac{V_{0}-E_{e\rho }}{\mu }}a\right)=-B_{1}J_{1}\left(\sqrt{E_{e\rho }}a\right)-Y_{1}\left(\sqrt{E_{e\rho }}a\right)$$
  with: $\left|\begin{array}{c} A_{1}=\frac{J_{0}\left(\sqrt{E_{e\rho }}b\right)Y_{0}\left(\sqrt{E_{e\rho }}a\right)+Y_{0}\left(\sqrt{E_{e\rho }}b\right)J_{0}\left(\sqrt{E_{e\rho }}a\right)}{I_{0}\left(\sqrt{\frac{V_{0}-E_{e\rho }}{\sigma }}a\right)J_{0}\left(\sqrt{E_{e\rho }}b\right)}\\ B_{1}=-\frac{Y_{0}\left(\sqrt{E_{e\rho }}b\right)}{J_{0}\left(\sqrt{E_{e\rho }}b\right)} \end{array}\right.$
- For $E_{e\rho }>V_{0}$
  (8) $$A_{2}\sqrt{\mu \left(1-\frac{V_{0}}{E_{e\rho }}\right)}J_{1}\left(\sqrt{\frac{V_{0}-E_{e\rho }}{\mu }}a\right)=B_{2}J_{1}\left(\sqrt{E_{e\rho }}a\right)+Y_{1}\left(\sqrt{E_{e\rho }}a\right)$$
  with: $\left|\begin{array}{c} A_{2}=\frac{J_{0}\left(\sqrt{E_{e\rho }}b\right)Y_{0}\left(\sqrt{E_{e\rho }}a\right)+Y_{0}\left(\sqrt{E_{e\rho }}b\right)J_{0}\left(\sqrt{E_{e\rho }}a\right)}{J_{0}\left(\sqrt{\frac{E_{e\rho }-V_{0}}{\sigma }}a\right)J_{0}\left(\sqrt{E_{e\rho }}b\right)}\\ B_{2}=-\frac{Y_{0}\left(\sqrt{E_{e\rho }}b\right)}{J_{0}\left(\sqrt{E_{e\rho }}b\right)} \end{array}\right.$

Next, we shall establish the electron energy levels taking into account the presence of the external applied magnetic field along the *z*-axis. In this case, it will be difficult to make the Schrödinger equation in a usual form whose solution is known. However, the use of a numerical method is strongly recommended. For a suitable choice of the trial wave function with the variational method, we performed another calculation using the finite element method (FEM) via Comsol Multiphysics software, which is a well known and established approach to solving the governing Partial Differential Equations (PDEs) numerically [35]. A good agreement is found between the two methods for the calculation of the electron ground state energy under the magnetic field effect (denoted $E_{0e}\left(B\right)$), according to the following approximate wave function:

(9) $$\psi_{e}\left(\rho_{e}\right)=f\left(\rho_{e}\right)e^{\left(-\lambda \rho_{e}^{2}\right)}$$

Then

(10) $$E_{0e}\left(B\right)=\min_{\lambda }\frac{<\psi_{e}\left(\rho_{e}\right)\left|H_{0e}\left(B\right)\right|\psi_{e}\left(\rho_{e}\right)>}{<\psi_{e}\left(\rho_{e}\right)|\psi_{e}\left(\rho_{e}\right)>}\;$$

where λ is the related variational parameter and $H_{0e}\left(B\right)$ is the single electron Hamiltonian with respect to the presence of the magnetic field $\vec{B}$. This last one is given by:

(11) $$H_{0e}\left(B\right)=\left\{\begin{array}{c} -\mu \nabla^{2}+\mu \gamma L_{z}+\frac{\mu }{4}\gamma^{2}\rho_{e}^{2}+V_{0},\;\mathrm{if}\;0<\rho_{e}<a\\ -\nabla^{2}+\gamma L_{z}+\frac{1}{4}\gamma^{2}\rho_{e}^{2},\;\mathrm{if}\;a<\rho_{e}<b \end{array}\right.$$

After this detailed calculation, we can now calculate the ground state energy of the electron-donor impurity system $E_{eD}\left(B\right)$ by minimizing the expectation value of $H_{eD}$ given in Equation (3), with respect to the variational parameters $\lambda_{1}$ and $\lambda_{2}$:

(12) $$E_{eD}\left(B\right)=\min_{\lambda_{1},\lambda_{2}}\frac{<\psi_{eD}\left({\vec{r}}_{eD}\right)\left|H_{eD}\right|\psi_{eD}\left({\vec{r}}_{eD}\right)>}{<\psi_{eD}\left({\vec{r}}_{eD}\right)|\psi_{eD}\left({\vec{r}}_{eD}\right)>}$$

In the following, the calculated photoionization cross-section can be used to describe the process of removing an electron from the impurity atom that is embedded in the core material. The impurity can be excited by the incident photon, which can then lead to the removal of an electron from the impurity. It is usually denoted by the symbol σ and has units of square meters ($m^{2}$). In the dipole approximation, it is given by the expression [36]:

(13) $$\sigma \left(\hbar \;\omega \right)=\frac{4\;\pi^{2}\;\alpha_{FS}}{n_{r}}{\left(\frac{F_{eff}}{F_{0}}\right)}^{2}{\left(\frac{m_{e}^{*}}{m_{0}}\right)}^{2}\hbar \;\omega \sum_{f}{\left|\langle \Psi_{i}|\vec{\zeta }.\vec{r_{e}}|\Psi_{f}\rangle \right|}^{2}\delta \left(E_{f}-E_{i}-\hbar \;\omega \right)$$

where $\alpha_{FS}=\frac{e^{2}}{\hbar \;c}$ is the fine structure constant, ℏω is the incident photon energy, $n_{r}$ is the refractive index of semiconductors, $F_{eff}/F_{0}$ is the ratio of effective electric field of the incoming photon to the average electric field in the medium. Since the ratio is not known to affect the shape of the photoionization cross-section, it is generally set equal to unity. $\vec{\zeta }$ is the light wave polarization vector and $m_{0}$ is the free electron mass. $\langle \Psi_{i}|\vec{\zeta }.\vec{r_{e}}|\Psi_{f}\rangle$ is the matrix element between the initial and final states of the dipole moment of the impurity. $E_{f}$ and $E_{i}$ are the energies of the final and initial states, respectively. The final state wave function corresponds to $\psi_{e}\left(\rho_{e}\right)$, while the initial state corresponds to the donor impurity wave function $\psi_{eD}\left({\vec{r}}_{eD}\right)$. It is worth mentioning that we have considered the case where the polarization vector is contained in the xy-plane (perpendicular polarization), so due to the invariance of the system by rotation around the *z*-axis the polarization of the incident radiation may then be chosen in the *x*-direction. Finally, the Dirac delta function is usually substituted by a Lorentzian one via the following expression:

(14) $$\delta \left(E_{f}-E_{i}-\hbar \;\omega \right)=\frac{\Gamma }{{\left(E_{f}-E_{i}-\hbar \;\omega \right)}^{2}+\Gamma^{2}}\;$$

where Γ is the hydrogenic impurity linewidth taken as $0.1R_{D}^{*}$.

## 3. Results

Our theoretical study of Al${}_{x}$Ga${}_{1-x}$As/GaAs-based core/shell NWs with cylindrical geometry was carried out for the alloy concentration x=0.1. Our results are obtained using the following parameter values: Since the crystal structures and lattice constants of Al${}_{x}$Ga${}_{1-x}$As and GaAs are very closed, we expect negligible strain at the interface and the dielectric mismatch effect. The conduction band barrier height energy $V_{0}=Q_{c}\times 1.247x$ where the conduction band offset $Q_{c}$ is taken to be about 63% of the band gap differences between the two considered materials [37,38]. The dielectric constant in the core and shell region is $\varepsilon_{core}=13.18-3.12x$ and $\varepsilon_{shell}=13.18$, respectively. Several analyses have shown that the energy band gap can be expressed as a linear function of Al content *x* as $E_{g}\left(eV\right)=1.424+1.247x$ [39,40], also for the effective mass of the electron, which can be expressed in units of the free electron mass $m_{0}$ by the formula $m_{e}^{*}=0.063+0.083x$. To be within the size standards adopted for non-wires, we took throughout this study the following sizes: the shell radius $b=2a_{D}^{*}$ and the height $h=20a_{D}^{*}$. For GaAs material, the dimensioning units are: $a_{D}^{*}=11.088$ nm, $R_{D}^{*}=4.932$ meV and γ=1 corresponding to the external magnetic field B=5.3682 Tesla.

## 4. Discussion

**Figure 2:**
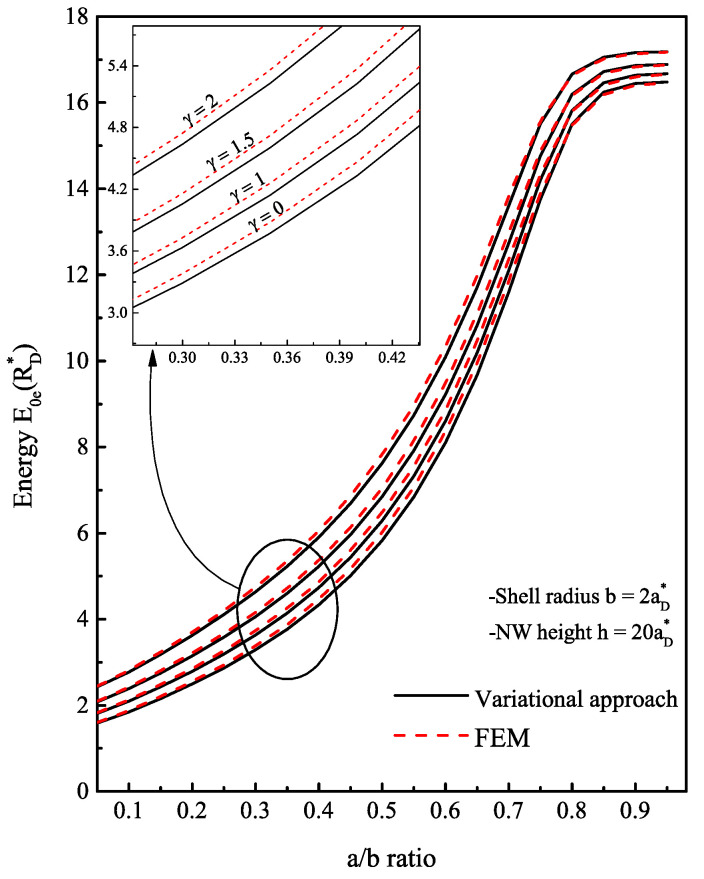
Electron ground state energy of Al${}_{x}$Ga${}_{1-x}$As/GaAs core/shell NW with x=0.1 calculating via variational method (solid line) according to Equation (10) and via FEM implemented in COMSOL Multiphysics (dashed line). The FEM is used by modeling the Schrödinger equation $H_{0e}\left(B\right)\psi_{e}\left(\rho_{e}\right)=E_{0e}\left(B\right)\psi_{e}\left(\rho_{e}\right)$ with Partial Differential Equations model. The results of the two methods are sufficiently consistent. The core radius was varied by the ratio (a/b) and three cases of the applied magnetic field are considered: γ=1,1.5 and 2.

Our first discussion concerns the validity of the trial wave function choice given in Equation (9) in order to later calculate the impurity ground state under the applied magnetic field. In terms of comparison, we plotted in Figure 2 the variation of the electron ground state as a function of the radii ratio a/b for different values of the magnetic field *B* using both the variational approach and FEM. As can be seen from the figure, the obtained values from both methods are very close, which confirms the good choice of the wave function that describes the localization of the electron inside the structure. First of all, for a small core radius (a→0), it is clear that the electron wave function strongly depends on the shell radius *b*. Therefore, the electron is trapped inside the shell and occupies an energy level near the minimum of the GaAs conduction band. As soon as the core radius becomes significant compared to the shell radius, the ground state level will have a higher energy, which remains lower than the conduction-band offset until a threshold value $a=a_{0}$, where the fundamental energy eventually becomes higher than $V_{0}$. We note that the value of $a_{0}$ is determined from the transcendental equation (Equation (7)), providing that the energy $E_{0e}\left(B\right)=V_{0}$. In our case, the barrier energy in units of $R_{D}^{*}$ is about 15.98 and it is assumed to be unaffected by $\vec{B}$. We can conclude from Figure 2 that the applied magnetic field gives rise to an additive energy term to the electron energy without changing the behavior according to the core/shell size. Thus, the electron moves much faster towards the shell than in the case of zero field (γ=0), leading to a slight reduction of the threshold value $a_{0}$.

**Figure 3:**
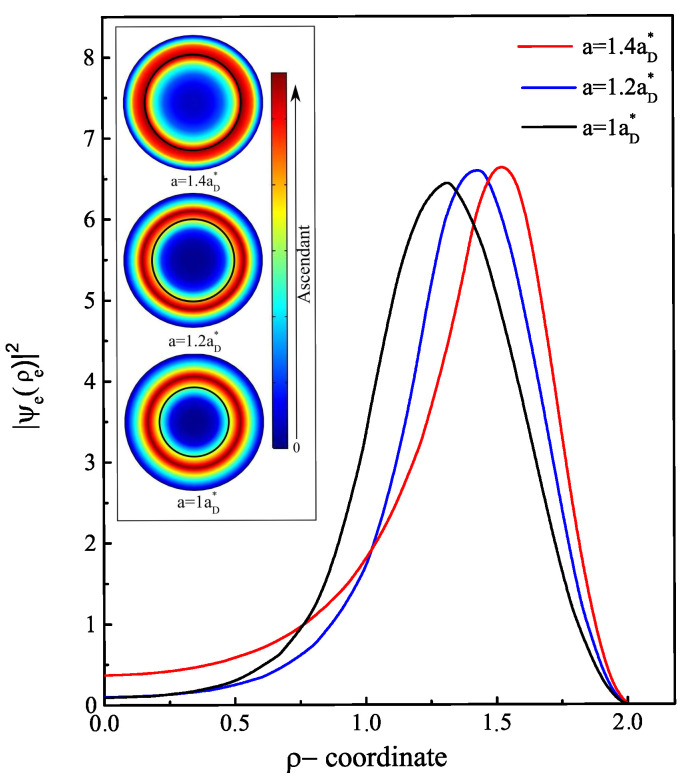
Electron probability density in Al${}_{x}$Ga${}_{1-x}$As/GaAs core/shell NW along the radial direction ρ at zero magnetic field (γ=0). Three core radius values are considered a=1,1.2 and $1.4a_{D}^{*}$ less than the threshold radius $a_{0}$. Each linear representation corresponds to the planar representation, which is the xy-plane cross-section of the electron density distribution at the middle of the cylinder.

**Figure 4:**
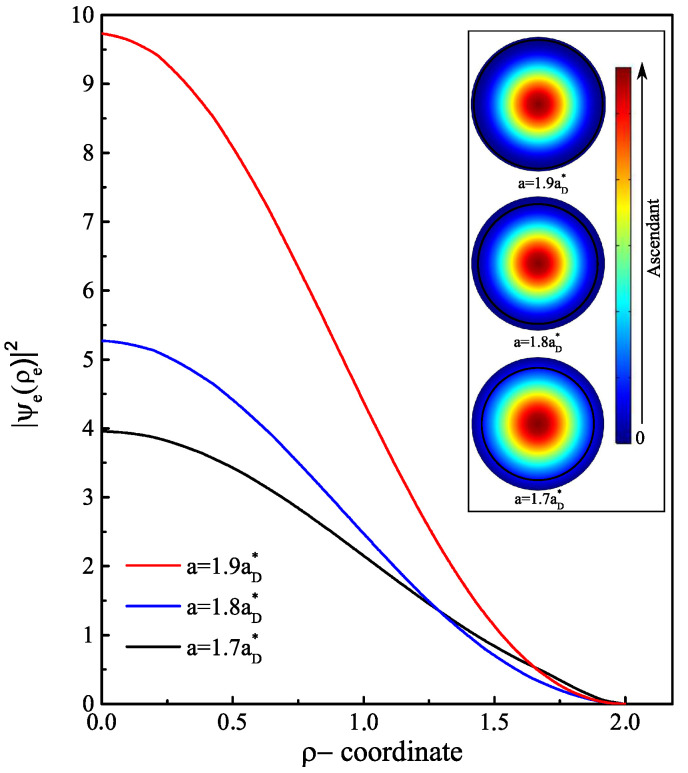
Electron probability density in Al${}_{x}$Ga${}_{1-x}$As/GaAs core/shell NW along the radial direction ρ at zero magnetic field (γ=0). Three core radius values are considered a=1.7,1.8 and $1.9a_{D}^{*}$ greater than the threshold radius $a_{0}$. Each linear representation corresponds to the planar representation, which is the xy-plane cross-section of the electron density distribution at the middle of the cylinder.

**Figure 5:**
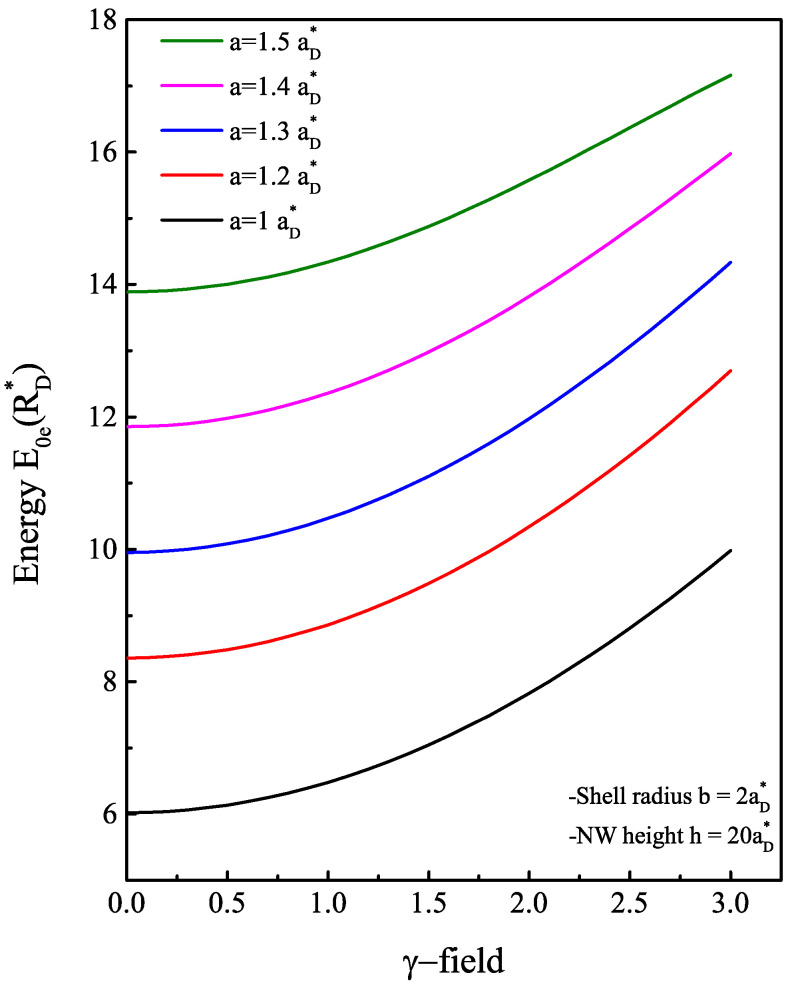
Variation of the electron ground state energy inside Al${}_{x}$Ga${}_{1-x}$As/GaAs-based core/shell NW, as a function of the magnetic field for different values of the core radius: from 1 by 0.1 to 1.5 $a_{D}^{*}$. The pace of evolution is semi-parabolic, the magnetic field has a remarkable effect only from an intensity γ≥1.

Contrary to an infinite confinement barrier, where the ground state energy increases without bound as a function of the a/b ratio [36,41], the energy $E_{0e}\left(B\right)$ begins to level off once the electron exits the shell region. With a finite confinement barrier, the electron has another mechanism of displacement inside the structure. Even with $E_{0e}\left(B\right)<V_{0}$, there is always some probability for the electron to penetrate into the core region through the tunneling effect. To estimate the probability of the electron’s presence in each region, we display the radial probability density of the electron for three values of $a<a_{0}$ in Figure 3 and three other values of $a>a_{0}$ in Figure 4. The probability density has a peak that represents the maximum probability of presence of the electron. In Figure 3, it can be observed that as long as the core radius stays lower than the radius threshold or the electron energy is less than the core barrier, the electron has a higher probability of being inside the GaAs material than the Al${}_{x}$Ga${}_{1-x}$As. When the core radius is close to $a_{0}$ (here $a_{0}=1.5885a_{D}^{*}$), the electron has an equal probability of being inside the core as well as the shell. Moreover, it is noteworthy that the electron continually leaks towards the core/shell interface. This may be predictable since there is an asymmetry of the applied confinement potential from each shell interface. Once the electron reaches a sufficient energy $E_{0e}\left(B\right)=V_{0}$, it starts to have a higher probability of penetrating towards the core side and heading towards the center as this area expands. Moreover, the results represented in Figure 4 show that from $a=1.7a_{D}^{*}$, the probability density becomes localized only at the core center with more pronounced peak amplitude as *a* values approach $b=2a_{D}^{*}$. This finding explains the stagnation of the electron ground state energy when the ratio of a/b tends to 1, as shown in Figure 2. The behavior of the electron ground state energy under the external magnetic field effect is depicted in Figure 5. The plot shows the curve of $E_{0e}\left(B\right)$ for three typical cases of core radius (a=1,1.2 and $1.3a_{D}^{*}$). After suitable fitting, we observed that this energy behaves as a quadratic polynomial function with the field $\vec{B}$. This is mainly due to the term of $\gamma^{2}$ in the Hamiltonian of the electron. For low values of the magnetic field (γ<1), we remark that the electron energy is not influenced much and it remains almost constant. Increasing the strength of the external magnetic field from γ=1, the electron reacts with the field lines by increasing its energy. Since no spin degree of freedom has been considered in this study, this increase in energy has only one explanation, which is that the application of intense magnetic fields creates an additive potential energy to the electron. On the other hand, this allows to reduce the threshold value of the core radius $a_{0}$ by the fact that the electron can reach the energy $E_{0e}\left(B\right)=V_{0}$. In our case, the threshold value becomes ($1.5a_{D}^{*}$) for a value of γ=2.2 or ($1.4a_{D}^{*}$) for γ=3. In the same way, another study showed that the barrier height $V_{0}$ decreases as the magnetic field increases as a result of variation of band offsets [42]; thus, the value of $a_{0}$ will be reduced.

**Figure 6:**
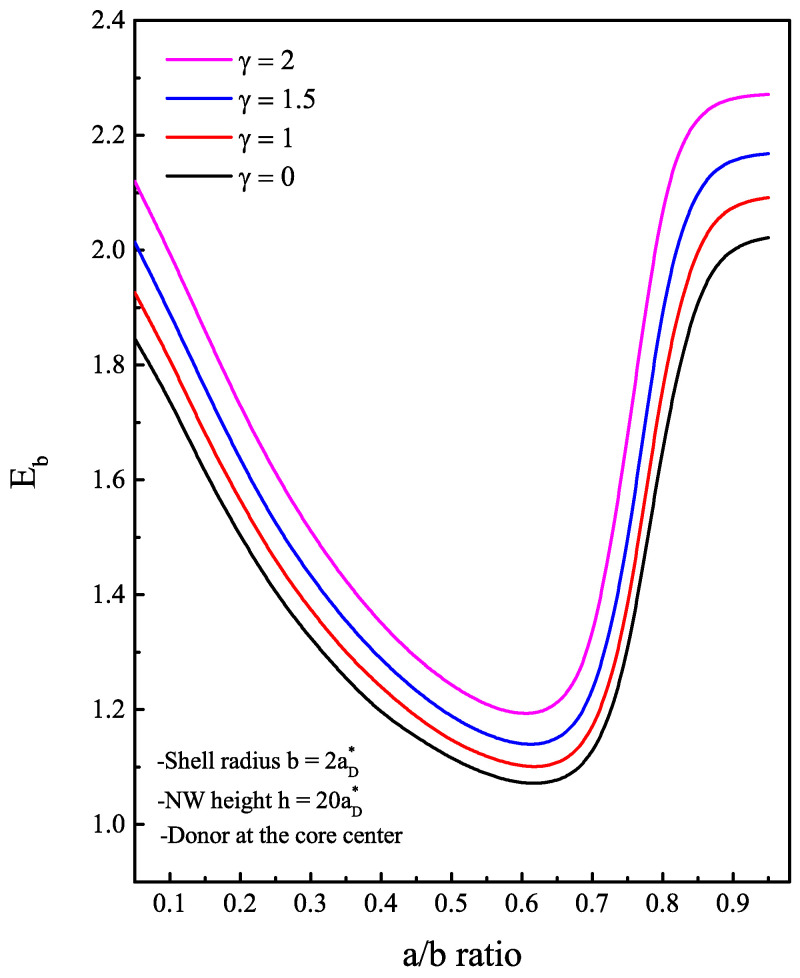
The calculated impurity binding energy as a function of the a/b ratio. The dimensionless measure γ of the magnetic field strength is varied from 1 by 0.5 to 2. The donor impurity is located at the core center along the *z*-axis.

**Figure 7:**
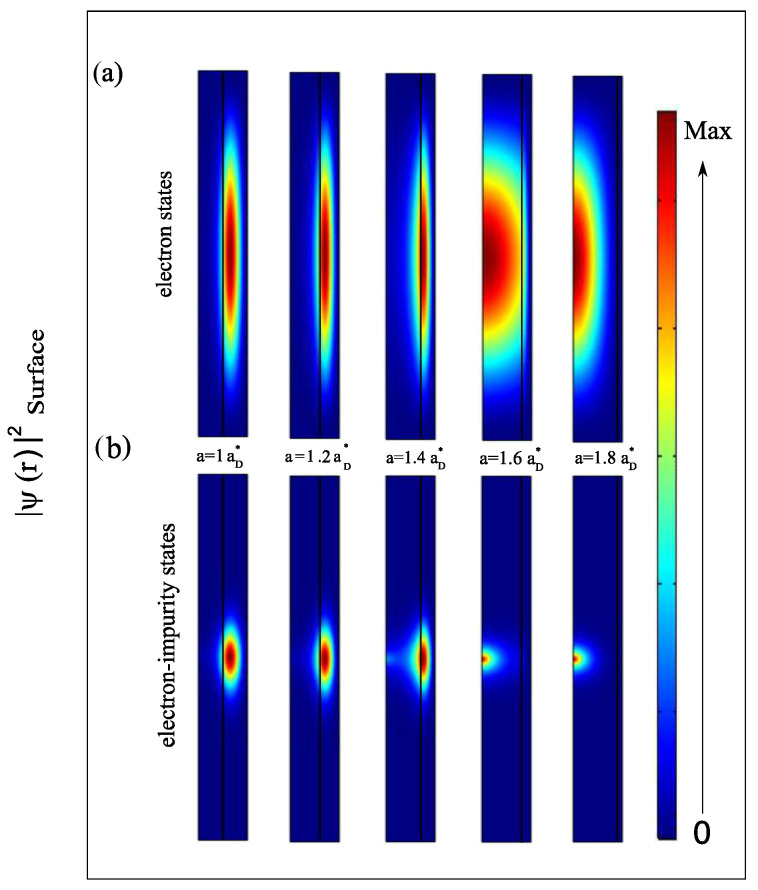
The probability density distribution of the electron inside Al${}_{x}$Ga${}_{1-x}$As/GaAs-based core/shell NW with shell radius $b=2a_{D}^{*}$ and height $h=20a_{D}^{*}$, for different values of the core radius: from $a=1a_{D}^{*}$ by 0.2 to 1.8$a_{D}^{*}$. The square of the wave function describes the ground state level of (**a**) single electron and (**b**) electron-donor impurity system. The representation is the ρz-plane cross-section with 2D axi-symmetric model.

This result can be further confirmed by analyzing the magnetic field’s effect on the binding energy of the donor impurity, which is defined as $E_{b}=E_{0e}\left(B\right)-E_{eD}\left(B\right)$. We assume that the impurity can be localized at the core center, so that the Coulomb interaction term is added to the electron Hamiltonian. In Figure 6, the binding energy is plotted as a function of the radii ratio a/b for different magnetic field values. The behavior of the binding energy can practically be interpreted with what was discussed in Figure 3 and Figure 4. The Coulomb interaction is the main responsible factor since $E_{b}$ is inversely proportional to the electron-impurity distance. Because the electron wave function is closed towards the inner side of the shell when $a<a_{0}$, the electron will move away from impurity every time the core radius increases. The quantum confinement effect enhances the electron energy and favors the penetration of wave functions in the core, leading to a strong impurity binding energy. The variation of the energy $E_{b}$ then presents a minimum, which corresponds to a large possible electron-impurity distance. This is the case where the electron begins to have an energy very close to the barrier one. As is known, increasing the strength of the external magnetic field enhances the electron ground state energy, bringing the electron closer to the interface while $E_{0e}\left(B\right)<V_{0}$ and then towards the core center once $E_{0e}\left(B\right)>V_{0}$, which justifies the increase in the binding energy. Interestingly, the minimum of the $E_{b}$ is slightly shifted towards lower a/b ratio values. The attraction of the electron towards the impurity can be seen clearly in the illustration of Figure 7 when we put in comparison the probability of finding the electron inside the structure without and with the presence of the donor impurity, (a) and (b), respectively. In general, the probability is proportional to the square of the wave function $\psi_{e}\left(\rho_{e}\right)$ for (a) and $\psi_{eD}\left({\vec{r}}_{eD}\right)$ for (b). The probability density profiles for energy levels of the ground state shows that, in the case of a single electron, the localization occupies a large section in the ρz-plane of 2D axi-symmetric view of the structure. However, in the presence of an on-center donor impurity, the cross-section of the probability density reduced as a result of the Coulombic interaction, which depends strongly on the quantum size effect.

**Figure 8:**
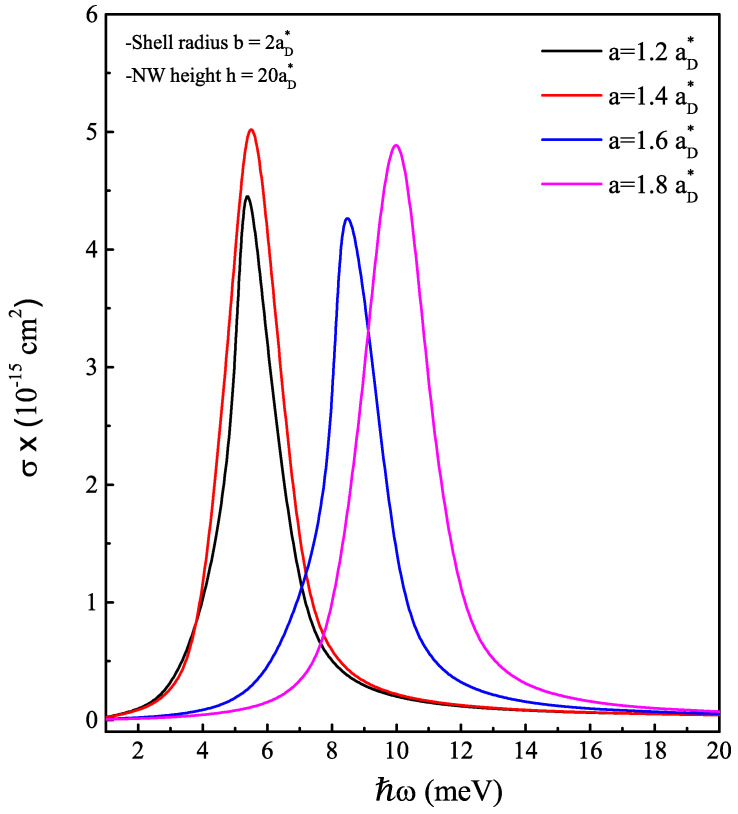
On-center donor impurity-related photoionization cross-section coefficient in cylindrical Al${}_{x}$Ga${}_{1-x}$As/GaAs-based core/shell NW as a function of the incident photon energy at zero magnetic field. The core radius varies from $1.2a_{D}^{*}$ by 0.2 to 1.8$a_{D}^{*}$.

**Figure 9:**
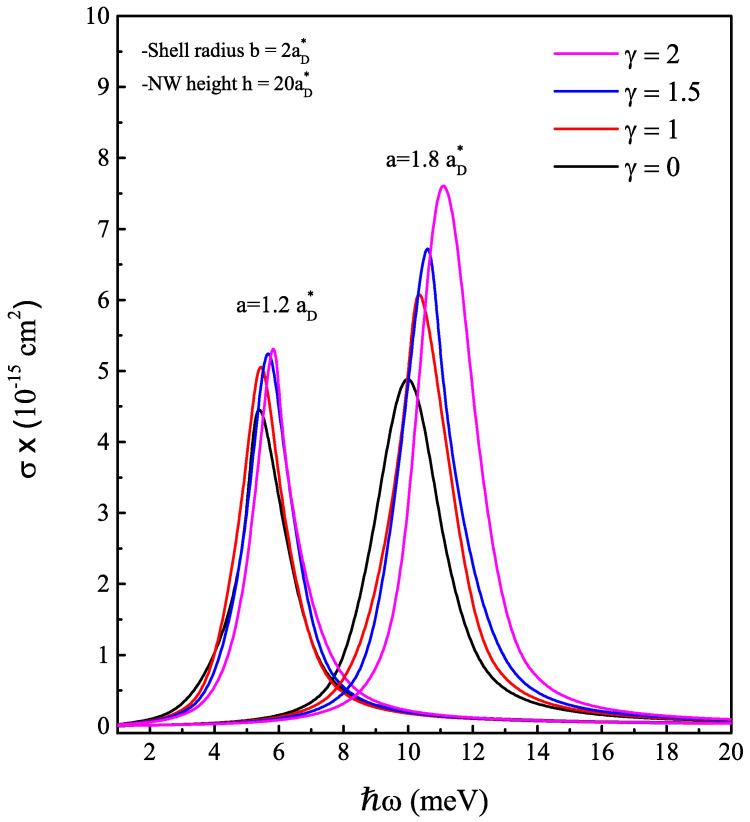
On-center donor impurity related photoionization cross-section coefficient in cylindrical Al${}_{x}$Ga${}_{1-x}$As/GaAs-based core/shell NW as a function of the incident photon energy for two typical core radiuses $a=1.2a_{D}^{*}$ and 1.8$a_{D}^{*}$. We take the following magnetic field strength: γ = 0, 1, 1.5 and 2.

As is known, the interaction between the electron and the impurity creates a localized state in the band gap of the semiconductor material. This gives the opportunity to investigate one of the optical properties—that of the impurity photoionization cross-section. The process of photoionization occurs when a photon interacts with an atom, molecule or solid and provides enough energy to remove an electron from the target material. In the context of impurities, photoionization cross-section can be used to describe the process of removing an electron from an impurity atom that is embedded in a host material. The impurity can be excited by the incident photon, which can then lead to the removal of an electron from the impurity. In the next figures, the coefficient σ calculated via Equation (13) is depicted as a function of the incident photon energy (ℏω) under the core radius effect (Figure 8) and the magnetic field effect (Figure 9). In both cases, the PCS behaves as a Lorentzian function, representing the probability transition of the electron from the impurity ground state level to a single electron ground state level at the conduction band. The resonance peak occurs when the energy of the incoming photon matches the energy required to excite the electron to realize this transition. We found that this energy is mainly related to the impurity binding energy. For this reason, the shift of the resonance peak towards high energies is obvious at once the core radius takes values greater than $1.4a_{D}^{*}$. For a fixed core radius, the increase of the external magnetic field also shifts the resonance peak position towards the highest photon energy, especially when the core radius value exceeds the threshold value $a_{0}=1.5885a_{D}^{*}$ in the ascending order. Furthermore, we can see that the absorbed photons refer to electromagnetic waves with frequencies ranging from 0.1 to 10 THz, which falls between the microwave and infrared regions of the electromagnetic spectrum. Overall, the optical responses of cylindrical Al${}_{x}$Ga${}_{1-x}$As/GaAs-based core/shell nanowires can make them promising candidates for use in terahertz and infrared optoelectronics devices.

## 5. Conclusions

In this paper, we aimed to provide underlying theoretical tools in order to analyze the behavior of charge carriers inside Al${}_{x}$Ga${}_{1-x}$As/GaAs-based core/shell NWs. The effective mass model was adopted with the finite confinement barrier to give the mathematical formalism allowing to investigate the electronic and optical properties. After an adequate choice of the trial wave function validated by comparison with the finite element method, our numerical calculations showed that except for the quantum tunneling, the penetration of the electron wave function in the core region will be held if the core radius value is greater than a threshold value $a_{0}$ to be determined, so that the electron ground state was extremely sensitive to the core radius values. We also showed that the application of a magnetic field along the *z*-axis of the structure enhances this energy and allows to reduce the threshold value $a_{0}$. We studied the optical properties through the interaction between the electron and a donor impurity assumed to be localized at the core center. We found that the impurity binding energy can be increased or decreased based on the core radius value and is increased if the magnetic field increases. The calculated photoionization cross-section proved that the resonance peak can have a remarkable shift towards high energy, once the core radius is greater than $1.4a_{D}^{*}$ or for an intense applied magnetic field. Overall, our results show that besides size control, wrapping NWs with another material offers additional degrees of tuning NW optoelectronic properties.

## Data Availability

The data presented in this study are available on reasonable request to the corresponding authors.
